# OmpK35/36 absence does not confer carbapenem-resistance alone nor ceftazidime-avibactam resistance with one *bla*_KPC-2_

**DOI:** 10.3389/fcimb.2026.1735799

**Published:** 2026-02-20

**Authors:** Susu Wu, Yinyin Yang, Wanyao Xiang, Jin Zhang, Xinhua Luo, Mengqiao Xu, Dakang Hu, Haifang Zhang

**Affiliations:** 1 Department of Laboratory Medicine, Taizhou Municipal Hospital (Taizhou University Affiliated Municipal Hospital), School of Medicine, Taizhou University, Taizhou, Zhejiang, China; 2 Taizhou Key Laboratory of Infection and Tumor Immunology, Taizhou, Zhejiang, China; 3 Endoscopy Center of Taizhou Hospital Affiliated to Wenzhou Medical University, Taizhou, Zhejiang, China; 4 Key Laboratory of Sepsis of Taizhou, Taizhou, Zhejiang, China; 5 Department of Clinical Laboratory, the Second Affiliated Hospital of Soochow University, Suzhou, Jiangsu, China

**Keywords:** carbapenem, ceftazidime-avibactam, *Klebsiella pneumoniae*, polymorphism, porin

## Abstract

**Objective:**

Investigate the genetic background of porin OmpK35/36 in *Klebsiella pneumoniae* and their influence on antimicrobial susceptibility, particularly carbapenems and ceftazidime-avibactam (CZA).

**Methods:**

1407 *K. pneumoniae* genomes in GenBank were selected for analyzing outer membrane protein-related genes through BLAST method, including *ompK35*, *ompK36*, *ompK26*, *ompK37*, *ompA*, *ompR*, and carbapenemase genes, including *bla*_KPC_, *bla*_VIM_, *bla*_IMP_, *bla*_NDM_, *bla*_OXA-48_. Using MEGA 11.0, OmpK35/36 and *ompK35/36* phylogenetic trees were built among serotypes K1 and K2 strains. Further, serotype K1 NTUH-K2044 and *bla*_KPC-2_ were used to construct mutants to elucidate impacts of OmpK35/36 on drug-resistance.

**Results:**

The rates of *ompK35*, *ompK36*, *ompK26*, *ompK37*, *ompA*, and *ompR* in *K. pneumoniae* strains were 97.5%, 99.3%, 99.5%, 99.4%, 99.9%, and 100.0% respectively. The sequence similarities of OmpK35/36 and *ompK35/36* were both over 90.0%. *K. pneumoniae* strains with abnormal *ompK35/36* presented higher rates of carbapenemase genes than those with normal *ompK35/36*. As to Δ*ompK36*, the minimum inhibitory concentrations (MICs) of piperacillin, cefoxitin, cefazolin, cefuroxime, and imipenem increased to 4, 4, 4, 8, and 4 times respectively compared with those against NTUH-K2044; the MICs of piperacillin, cefoxitin, cefazolin, cefuroxime, imipenem, and meropenem increased to 8, 32, 32, 16, 8, and 8 times in Δ*ompK35/36* respectively. The deletions of *ompK35/36*, especially the double deletion, would greatly help NTUH-K2044+*bla*_KPC-2_ induce resistance to certain β-lactams. Further, the absence of *ompK35/36* elevated the MIC of CZA against NTUH-K2044+*bla*_KPC-2_.

**Conclusions:**

Highly conserved *ompK35/36* are widely present in *K. pneumoniae* strains. The loss of OmpK35/36 confers increased resistance to certain β-lactams, with OmpK36 being dominant. Moreover, OmpK35/36 loss is a contributor but not a determinant in the formation of carbapenem-resistance under the absence of *bla*_KPC-2_, as well as in the formation of CZA-resistance with one *bla*_KPC-2_.

## 1 Introduction

*Klebsiella pneumoniae*, a Gram-negative bacterium of *Enterobacterale*, is ubiquitously distributed in natural environment. It is typically armed with a polysaccharide capsule along with type 1 and 3 fimbriae, but lacks flagella. As an opportunistic pathogen, *K. pneumoniae* is associated with a range of infections including endocarditis, and sepsis (Navon-Venezia et al., 2017). Hypervirulent *K. pneumoniae* (HvKP) strains can even cause fatal invasive syndromes such as necrotizing, lung abscess, pyogenic liver abscess, and endogenous endophthalmitis (Paczosa et al., 2016).

The first carbapenem-resistant *K. pneumoniae* (CRKP) strain was reported in the United States in 1996 (Yigit et al., 2008), which has become increasingly prevalent worldwide. At present, the proportion of carbapenem resistance in clinical *K. pneumoniae* strains is around 25.0% in China mainland (Li et al., 2023). CRKP commonly exhibits multidrug-resistance, including resistance to quinolones, aminoglycosides, and cephalosporins. These resistance profiles confer prolonged hospital stay, increased healthcare cost, and elevated mortality (Xu et al., 2017), which prompts the World Health Organization to classify CRKP as a priority pathogen.

The convergence of antimicrobial abuse and the continual evolution of *K. pneumoniae* have facilitated the emergence of superbugs with carbapenem resistance and hypervirulence (Martin and Bachman, 2018). In China mainland between 2015-2017, over 5.0% of carbapenem-resistant isolates demonstrated the dual risks, with a rising trend (Zhang et al., 2020). This ‘superbug’ poses a formidable challenge for clinicians. Drug resistance may be developed by *K. pneumoniae* through various mechanisms, such as hydrolytic enzymes, loss of outer membrane proteins (OMPs), over-expression of efflux pumps, topoisomerases, lipopolysaccharide modifications and so on (Hennequin and Robin, 2016; Aguilar et al., 2025; Uehara et al., 2025b). Among these, the absence or reduced expression of the porins OmpK35 and OmpK36 significantly restricts the penetration of carbapenems into the cell, thereby conferring resistance (Zhang et al., 2015; Park et al., 2025; Xie et al., 2025). There are also studies indicating that mutations, defects, or loss of function in OmpK35/36, if further combined with KPC or CTX-M, may even lead to an increase in the minimum inhibitory concentration (MIC) of ceftazidime-avibactam (CZA) (Nelson et al., 2017; Shen et al., 2017; Castanheira et al., 2020; Gaibani et al., 2020; Zhao et al., 2024).

OmpK35 and OmpK36 are major trimeric β-barrel porins in the outer membrane of *K. pneumoniae* and facilitate the passive diffusion of small hydrophilic molecules, including nutrients and certain antibiotics like β-lactams (Brunson et al., 2019). They share structural and functional similarities with OmpF and OmpC in *Escherichia coli* respectively, and play critical roles in maintaining membrane integrity alongside peptidoglycan and lipopolysaccharide layers (Sugawara et al., 2016; Davin-Regli et al., 2024). However, despite their importance, a systematic review of OmpK35/36 impact on *K. pneumoniae* lacks.

To address this gap, an epidemiological analysis of OmpK35/36 sequences available in the GenBank database was conducted in this paper. Furthermore, the serotype K1 strain NTUH-K2044 was used to elucidate the impacts of OmpK35/36 on its drug-resistance, particularly resistance to carbapenems and CZA.

## 2 Materials and methods

### 2.1 Strains, plasmids, and primers

A total of 1407 *K. pneumoniae* genomes (**Supplementary Table S1**) were downloaded from GenBank (https://www.ncbi.nlm.nih.gov/datasets/genome/?Taxon=573; as of August 30, 2022). K1 *K. pneumoniae* strain NTUH-K2044 is a typical strain of hypervirulence with accession number AP006725.1. It belongs to sequence type 23. HS11286 is a typical CRKP with accession number CP003200.1. It belongs to sequence type 11 and contains *bla*_KPC-2_ on the plasmid pKPHS1. The plasmids pCAS (apramycin resistance, used for gene deletion), pSGKP (rifampicin resistance, used for gene knockout), and pBAD24 (apramycin resistance, used for gene complementation) are from the authors’ medical laboratory. The competent DH5α is a product of Shanghai Yisheng Company, China.

The primers used in this study are shown in **Table 1**.

**Table 1:** Primers used in this study.

Primer	Sequence (5'-3')	Purpose
*ompK35* up F	GTCACCGGCGTGCAGAACGT	*ompK35* upstream homologous arm
*ompK35* up R	TATTTATTACCCTCATTAAT	
*ompK35* down F	ATTAATGAGGGTAATAAATATCTGCAGTACACCCCTTCGT	*ompK35* downstream homologous arm
*ompK35* down R	AGTATGCGTATTCGCGCACT	
*ompK36* up F	GAAACCGCGCCGGGAAGGTT	*ompK36* upstream homologous arm
*ompK36* up R	GTTATTAACCCTCTGTTTGT	
*ompK36* down F	ACAAACAGAGGGTTAATAACGTTGCAAGCTGCATAACAAA	*ompK36* downstream homologous arm
*ompK36* down R	CTCTGATTAATAACCTGCAG	
*ompK35-N20-g* F	GGTactagtACCGTTGTCGAACGCGTAGGGTTTTAGAGCTAGAAATAGCAAGTT	g-RNA fragment of *ompK35*
*gRNA* R	GCCGCTCTAGAAGTAGTGGA	
*ompK36-N20-g* F	GGTactagtGCCGAATTCCGGCAGAACGTGTTTTAGAGCTAGAAATAGCAAGTT	g-RNA fragment of *ompK36*
gRNA R	GCCGCTCTAGAAGTAGTGGA	
*c-ompK35* F	CTCCATACCCGTTTTTTTGGATGATGAAGCGCAATATTCT	*ompK35* complement sequence
*c-ompK35* R	TCTCATCCGCCAAAACAGCCTTAGAACTGGTAAACGATAC	
*c-ompK36* F	CTCCATACCCGTTTTTTTGGATGAAAGTTAAAGTACTGTC	*ompK36* complement sequence
*c-ompK36* R	TCTCATCCGCCAAAACAGCCTTAGAACTGGTAAACCAGGC	
*c-ompK35/36 F*	GTATCGTTTACCAGTTCTAAATGAAAGTTAAAGTACTGTC	*ompK35/36* complement sequence
*c-ompK35/36 R*	TTAGAACTGGTAAACGATAC	
pBAD24 F	GGCTGTTTTGGCGGATGAGA	Plasmid pBAD24 sequence
pBAD24 R	CCAAAAAAACGGGTATGGAG	
*ompK35* F(YZ)	CTCCAGCTCTAACCGTAGCG	internal sequence of *ompK35* gene
*ompK35* R(YZ)	CAGCCGCTTTGGTGTAATCG	
*ompK36* F(YZ)	CAGGCCTGAAATTTGGCGAC	*ompK36* internal sequence
*ompK36* R(YZ)	TCGTCGGTACGTTTGGAGTG	
*ompK35* F(CX)	TTACCCGCACATCTTGC	*ompK35* bidirectional sequencing
*ompK35* R(CX)	ATCGATGCCCAGATAGTTT	
*ompK36* F(CX)	TTTCCCTGACCATTTTGCGG	*ompK36* bidirectional sequencing
*ompK36* R(CX)	GGAGTGGTAGCTGAATCGCA	
*ompK35*-RT F	TATGCGGCCGTCATGTACTC	*ompK35* RT-PCR primers
*ompK35*-RT R	GGTCTGTACGTAGCCGATGG	
*ompK36*-RT F	CAACCTACCGTAACTCTGAT	*ompK36* RT-PCR primers
*ompK36*-RT R	GATGCCATCCCAAATATCGT	
*ompK26*-RT F	CGCTCGCTATCGCTATGAGT	*ompK26* RT-PCR primers
*ompK26*-RT R	GCTGCGGGCATAGACATAGT	
*ompK37-*RT F	GGCGACTCCTACACCTATGC	*ompK37* RT-PCR primer
*ompK37-*RT R	GCGTTCTGGCCTTCGTTTTT	
*ompR-*RT F	GAGCGTTATCTGACCGAGC	*ompR* RT-PCR primers
*ompR-*RT R	AGAGAGACCATCTTCGCCC	
16S rRNA F	TACCGCATAACGTCGCAAGA	internal reference
16S rRNA R	TTCCAGTGTGGCTGGTCATC	
*bla*_KPC-2_ F(YZ)	TCGCTAAACTCGAACAGG	internal sequence of *bla*_KPC-2_
*bla*_KPC-2_ R(YZ)	TTACTGCCCGTTGACGCCCAATCC	
*p-rmpA* F(YZ)	GAGTAGTTAATAAATCAATAGCAAT	*p-rmpA* internal sequence of *p-rmpA*
*p-rmpA* R(YZ)	CAGTAGGCATTGCAGCA	

RT-PCR, Real time fluorescence quantitative PCR.

### 2.2 Genetic backgrounds of *ompK35/36*

Based on the gene sequences of NTUH-K2044 and the standard sequences of carbapenemase gene (**Supplementary Table S2**), the distribution of OMP-related genes including *ompK35*, *ompK36*, *ompK26*, *ompK37*, *ompA*, *ompR*, and carbapenemase genes including *bla*_KPC_, *bla*_VIM_, *bla*_IMP_, *bla*_NDM,_*bla*_OXA-48_ in *K. pneumoniae* was predicted through the NCBI-BLAST website (https://blast.ncbi.nlm.nih.gov/Blast.cgi?Program=blastn&PAGETYPE=BlastSearch&LINK_LOC=blasthome) (coverage ≥ 80% and similarity ≥ 80%), and the similarities of *ompK35/36* sequences were compared. Then the serotype K1 and K2 were determined through the Pasteur website (https://bigsdb.pasteur.fr/cgi-bin/bigsdb/bigsdb.pl?db=pubmlst_klebsiella_seqdef&page=sequenceQuery). Phylogenetic trees of *omp35/36* and OmpK35/36 sequences from all K1/2 strains were built by MEGA 11.0 using the Neighbor Joining method and Bootstrap method (repeated 1000 times).

### 2.3 Knockout of *ompK35/36* in NTUH-K2044

On the basis of the wild strain NTUH-K2044, CRISPR-Cas9 gene deletion method (Hao et al., 2020) was utilized to construct such variants: Δ*ompK35*, Δ*ompK36*, and Δ*ompK35/36*. Primers *ompK35* up F, *ompK35* up R, *ompK35* down F and *ompK35* down R (**Table 1**) were used to construct the homologous fragment of *ompK35* while *ompK36* up F, *ompK36* up R, *ompK36* down F and *ompK36* down R (**Table 1**) were used to construct that of *ompK36*.

NTUH-K2044 was cultured to logarithmic growth phase in Luria-Bertani (LB) broth under 37°C and aerobic condition. The suspensions were then centrifuged at 4°C and 14000 round per minute for 10 minutes. The pallets were washed with 10% glycerol (Sigma, St. Louis, MO, USA) and re-dispersed gently at 4°C. After such triplicate washing, the 100 μl of re-dispersed NTUH-K2044 was electrotransformed with 5.0 μl of pCAS plasmid. The transformant was screened on LB agar plates with 50 μg/ml apramycin at 30°C, followed by regular PCR and electrophoresis.

Subsequently, competent cells with the pCAS plasmid were prepared, and electrotransformation of the constructed pSGKP plasmid (Primers: *ompK35-N20-g* F, *ompK36-N20-g* F, and *gRNA* R; **Table 1**) and clone verification were conducted again. Finally, the pCAS and pSGKP plasmids were eliminated using LB agar plates with 50% sucrose at 37°C, and verification was performed once more. The successful knockouts were confirmed through regular PCR, electrophoresis, and sequencing.

### 2.4 Complementation of *ompK35/36*

Fragments of *ompK35*, *ompK36*, *ompK35/36*, and pBAD24 plasmid were amplified using high-fidelity enzyme (NEB, MA, USA) according to the manufacturer’s protocol. Construction of pBAD24+*ompK35* (primers: pBAD24 F, pBAD24 R, *c-ompK35* F, and *c-ompK35* R), pBAD24+*ompK36* (primers: pBAD24 F, pBAD24 R, *c-ompK36* F, and *c-ompK36* R), and pBAD24+*ompK35/36* (primers: pBAD24 F, pBAD24 R, *c-ompK35/36 F*, and *c-ompK35/36* R) were performed using NEBuilder (NEB, MA, USA) and electrotransformed into competent DH5α cells. The successful clones were screened using LB agar plates with 100 μg/ml rifampin at 37°C. The harvested pBAD24+*ompK35*, pBAD24+*ompK36*, and pBAD24+*ompK35/36* were then electrotransformed in their corresponding mutants: Δ*ompK35*, Δ*ompK36*, and Δ*ompK35/36*. The complementation was confirmed using regular PCR, electrophoresis, and sequencing (**Supplementary Figure S1**).

### 2.5 Expressions of *ompK35*, *ompK36*, *ompK26*, and *ompK37*

The tested strains were cultured to logarithmic growth phase in LB broth under 37°C and aerobic condition. Total RNAs of tested strains were extracted using test kit (MolPure® Bacterial RNA Kit) (Yisheng, Shanghai, China) and reversely transcribed into cDNA using test kit (Hifair® V one-step RT-gDNA digestion SuperMix for qPCR) (Yisheng, Shanghai, China). Real-time quantitative PCR was conducted on an Applied Biosystems 7500 system to evaluate the expressions of *ompK35*, *ompK36*, *ompK26*, and *ompK37*, with 16S rRNA being the reference gene (**Supplementary Table S3**-**Supplementary Table S5**) (Primers: *ompK35*-RT F, *ompK35*-RT R, *ompK36*-RT F, *ompK36*-RT R, *ompK26*-RT F, *ompK26*-RT R, *ompK37*-RT F, *ompK37*-RT R, 16S rRNA F, and 16S rRNA R) (**Table 1**). The analysis was performed in line with the manufacturer’s protocol of the SYBR Green qPCR Mix (catalogue number: FS-Q1002; FOREVER STAR, Beijing, China).

### 2.6 Conjugation assay

NTUH-K2044+pCAS and HS11286 were chosen as the recipients and donors respectively. Both donors and recipients were cultured to logarithmic phase at 30°C. Further, 200 μl of donor cells and 800 μl of recipient cells were mixed and inoculated on the LB agar plate at 30°C overnight. Next, the transconjugants were selected with the meropenem (4 μg/ml) and apramycin (100 μg/ml). The transconjugants was determined by PCR using *bla*_KPC-2_ and *p-rmpA* as marker genes [Primers: *bla*_KPC-2_ F(YZ), *bla*_KPC-2_ R(YZ), *p-rmpA* F(YZ), and *p-rmpA* R(YZ)] (**Table 1**). The yielded transconjugant was NTUH-K2044+pCAS +*bla*_KPC-2_. HS11286 also served the donor to yield Δ*ompK35*+*bla*_KPC-2_, Δ*ompK36*+*bla*_KPC-2_, and Δ*ompK35/36*+*bla*_KPC-2_.

### 2.7 Antimicrobial susceptibility tests

The broth microdilution (Kangtai, Wenzhou, China) method was performed to determine the MICs of various antibiotics against knockout and complementation mutants of NTUH-K2044 (**Supplementary Table S6**), as shown in **Tables 2**, **3** (CZA). The experiments were conducted in accordance with the latest CLSI M100 (35^th^ edition). VITEK-2 compact (bioMérieux, Marcy l’-Étoile, France) and GN335 card (**Supplementary Table S7**) were used to determine the MICs of various antibiotics against the transconjugants, as shown in **Table 3**. Antimicrobial susceptibility tests were all performed in triplicate.

**Table 2:** MICs of antimicrobials against knockouts and complemented mutants of NTUH-K2044.

Antimicrobials	MICs (mg/L)							
	NTUH-K2044	Δ*ompK35*	Δ*ompK36*	Δ*ompK35/36*	Δ*ompK35* +*ompK35*	Δ*ompK36* +*ompK36*	Δ*ompK35/36* +*ompK35/36*
Piperacillin	2; S	4; S	8; S	16; SDD	2; S	4; S	16; SDD
Cefoxitin	1; S	2; S	4; S	32; R	2; S	4; S	16; I
Cefazolin	0.5; S	0.5; S	2; S	16; S	0.5; S	2; S	8; S
Cefuroxime	0.5; S	1; S	4; S	8; S	1; S	2; S	4; S
Ceftazidime	<0.125; S	0.125; S	<0.125; S	0.125; S	0.125; S	<0.125; S	0.125; S
Cefepime	<0.064; S	<0.064; S	0.064; S	0.125; S	<0.064; S	0.064; S	0.125; S
Cefotaxime	<0.125; S	<0.125; S	<0.125; S	0.125; S	<0.125; S	<0.125; S	0.125; S
Ceftriaxone	<0.064; S	<0.064; S	<0.064; S	0.064; S	<0.064; S	<0.064; S	0.064; S
Imipenem	0.064; S	0.125; S	0.25; S	0.5; S	0.125; S	0.25; S	0.25; S
Meropenem	<0.016; S	<0.016; S	0.016; S	0.064; S	<0.016; S	0.016; S	0.064; S
Amikacin	0.5; S	0.5; S	0.5; S	0.5; S	0.25; S	0.25; S	0.5; S
Ciprofloxacin	0.016; S	0.016; S	0.016; S	0.032; S	0.016; S	0.016; S	0.032; S
Chloramphenicol	4; S	8; S	4; S	8; S	4; S	4; S	4; S
Tetracycline	1; S	2; S	1; S	2; S	1; S	1; S	2; S
Polymyxin B	0.5; I	0.5; I	0.5; I	0.5; I	0.5; I	0.5; I	0.5; I

S, susceptible; I, intermediate; SDD, susceptible-dose-dependent; R, resistant.

**Table 3:** MICs of different antimicrobials against transconjugants.

Antimicrobials	MICs (mg/L)			
	NTUH-K2044+*bla*_KPC-2_	Δ*ompK35*+*bla*_KPC-2_	Δ*ompK36*+*bla*_KPC-2_	Δ*ompK35/36*+*bla*_KPC-2_
Ticarcillin-clavulanic acid	≥128/2; R	≥128/2; R	≥128/2; R	≥128/2; R
Piperacillin-tazobactam	≥128/4; R	≥128/4; R	≥128/4; R	≥128/4; R
Ceftazidime	4; S	16; R	16; R	16; R
Cefoperazone-sulbactam	16/8; S	16/8; S	≥64/32; R	≥64/32; R
Cefepime	0.5; S	1; S	2; S	≥32; R
Aztreonam	2; S	8; I	16; R	≥64; R
Imipenem	8; R	8; R	8; R	≥16; R
Meropenem	≥16; R	≥16; R	≥16; R	≥16; R
Amikacin	≤2; S	≤2; S	≤2; S	≤2; S
Tobramycin	≤1; S	≤1; S	≤1; S	≤1; S
Ciprofloxacin	≤0.25; S	≤0.25; S	≤0.25; S	≤0.25; S
Levofloxacin	≤0.12; S	≤0.12; S	≤0.12; S	≤0.12; S
Doxycycline	1; S	1; S	1; S	1; S
Tigecycline	≤0.5; S	≤0.5; S	≤0.5; S	≤0.5; S
Colistin	≤0.5; I	≤0.5; I	≤0.5; I	≤0.5; I
Trimethoprim-sulfamethoxazole	≤2/38; S	≤2/38; S	≤2/38; S	≤2/38; S
*Ceftazidime-avibactam	0.25/4; S	0.5/4; S	0.5/4; S	1/4; S

S, susceptible; I, intermediate; R, resistant.

### 2.8 Statistical analysis

The data analysis was conducted using GraphPad Prism 10.0 between two or more groups. The difference was considered significant while *p* < 0.05. Chi square test, one-way ANOVA, and two-way ANOVA were employed in statistics.

## 3 Results

### 3.1 The genetic backgrounds of *ompK35/36* in *K. pneumoniae* strains

**Figure 1:**
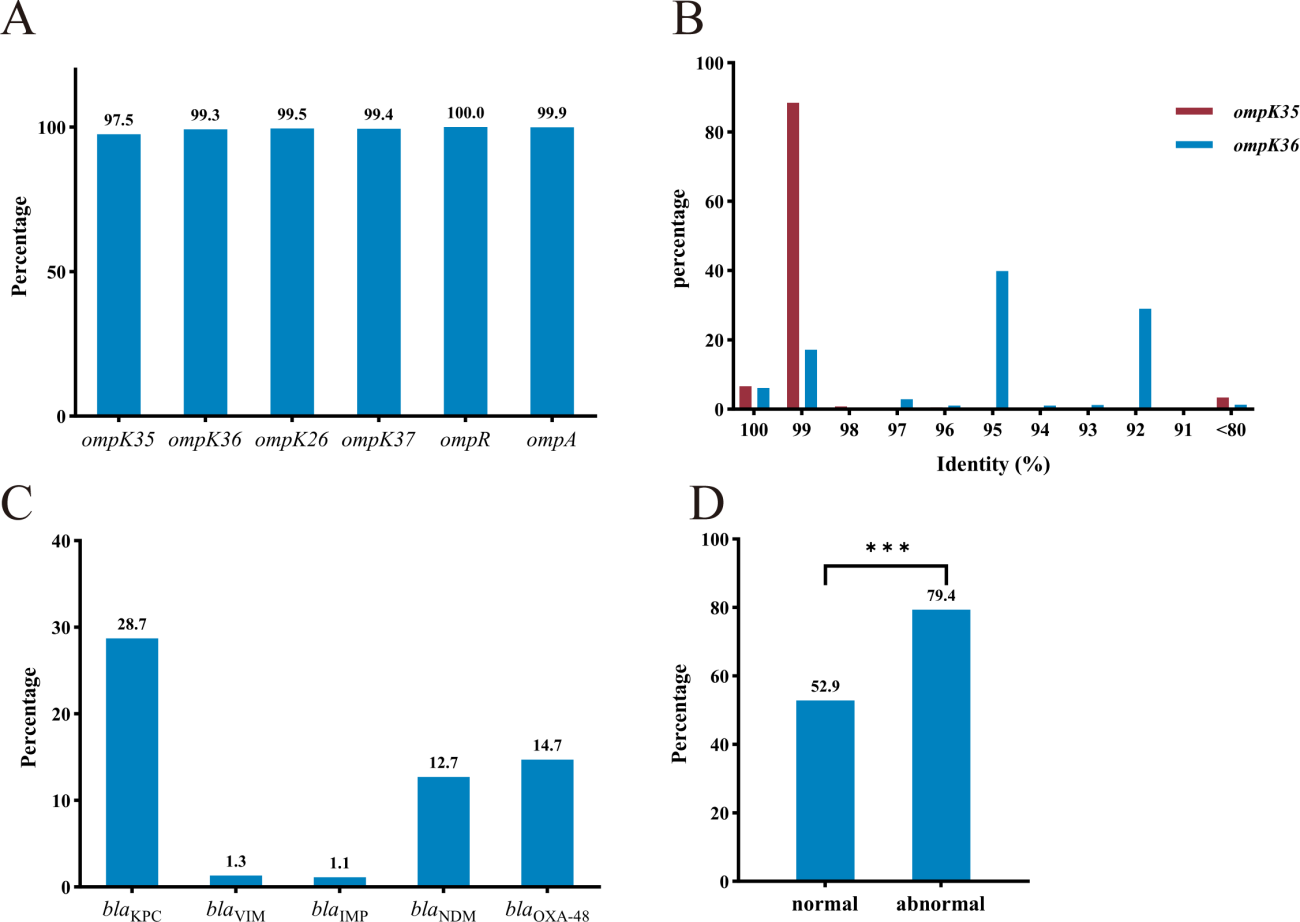
Genetic backgrounds of *ompK35/36* in *K. pneumoniae* strains. **(A)** Distribution of OMP-related genes; **(B)** Distribution of similarity percentages of *ompK35/36*; **(C)** Distribution of carbapenemase genes; **(D)** Percentages of carbapenemase genes in *K. pneumoniae* strains harboring normal and abnormal *ompK35/36* In **(D)**, normal group included 1346 strains while abnormal group included 61 strains; Chi square test was used. ***p < 0.001.

Among the 1407 K*. pneumoniae* genomes from the GenBank database, the positive rates of OMP-related genes *ompK35*, *ompK36*, *ompK26*, *ompK37*, *ompA*, and *ompR* were 97.5%, 99.3%, 99.5%, 99.4%, 99.9%, and 100.0%, respectively (**Figure 1A**). In order to further determine the similarity of *ompK35/36* sequences in 1407 K*. pneumoniae* strains, they were compared based on the *ompK35*/*36* sequences of NTUH-K2044. As shown in **Figure 1B**, similarity percentage of *ompK35* was mainly distributed at 99%, while those of *ompK36* were majorly at 95% and 92%. Among all the 1407 *K. pneumoniae* strains, 61 strains were found with abnormal *ompK35/36* sequences, including insertions, truncations, deletions and so on, as shown in **Figure 1B** (identity < 80%). The rates of carbapenemase genes *bla*_KPC_, *bla*_VIM_, *bla*_IMP_, *bla*_NDM_, and *bla*_OXA-48_ in *K. pneumoniae* strains were 28.7%, 1.3%, 1.1%, 12.7%, and 14.7% respectively (**Figure 1C**). As shown in **Figures 1D**, *K. pneumoniae* strains with abnormal *ompK35/36* sequences were found to present a significantly higher rate of the aforementioned carbapenemase genes.

### 3.2 Homologies of *ompK35/36* and OmpK35/36 in K1/2 *K. pneumoniae* strains

**Figure 2:**
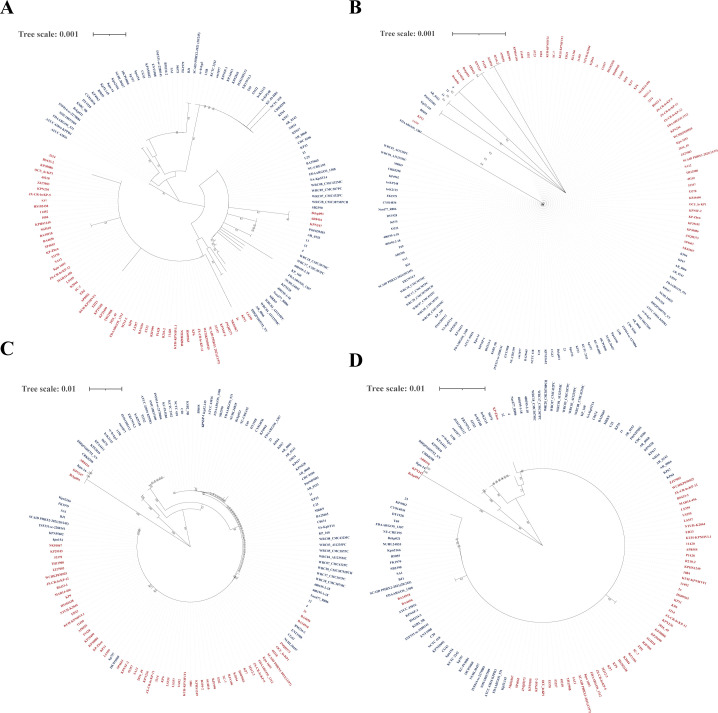
Homologies of *ompK35/36* and OmpK35/36 in K1/2 *K. pneumoniae* strains. **(A)** The phylogenetic tree of *ompK35* in K1/2 *K. pneumoniae* strains; **(B)** The phylogenetic tree of OmpK35 in K1/2 *K. pneumoniae* strains; **(C)** The phylogenetic tree of *ompK36* in K1/2 *K. pneumoniae* strains; **(D)** The phylogenetic tree of OmpK36 in K1/2 *K. pneumoniae* strains; Each strain name is plotted and K1 strains are shown in red while K2 strains are in blue. The analysis software is MEGA 11.0.

Among the 1407 K*. pneumoniae* strains, 65 K1 and 80 K2 strains were identified. To further analyze the similarity of OmpK35/36 between K1/2 strains, comparison of *ompK35/36* and OmpK35/36 were both conducted. As **Figure 2** shows, both *ompK35/36* and OmpK35/36 presented a high degree of consistency between K1/2 strains, especially OmpK35.

### 3.3 OMP-related genes expressions in NTUH-K2044

**Figure 3:**
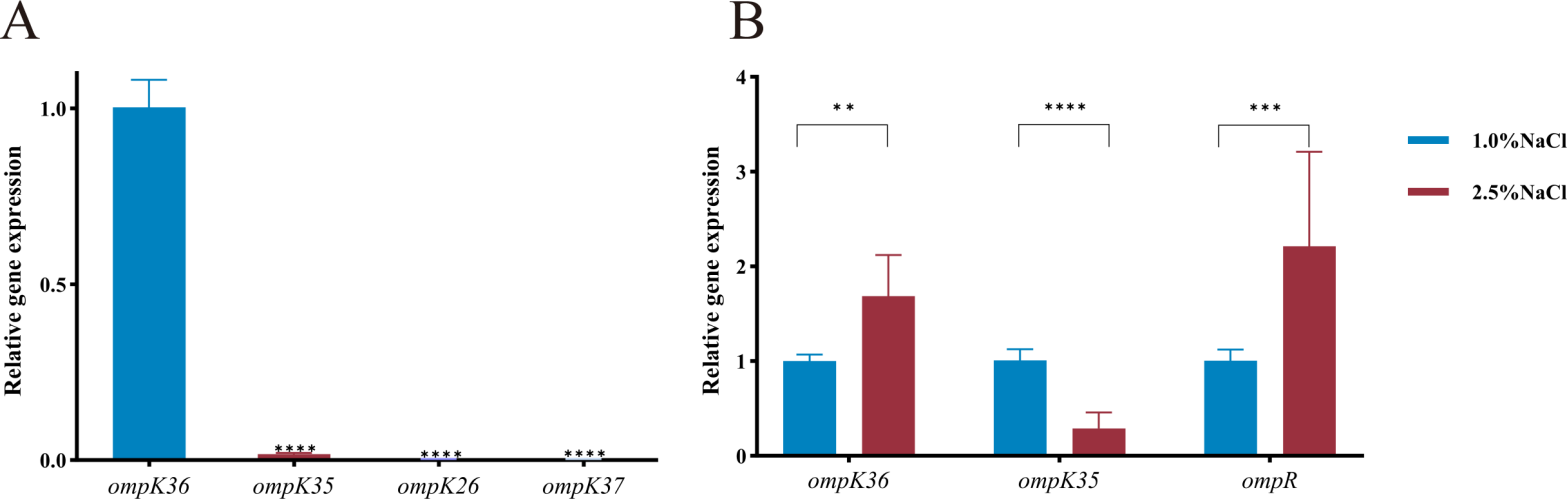
OmpK-related genes expression in NTUH-K2044. **(A)** Expression of *ompK36, ompK35, ompK26*, and *ompK37* in NTUH-K2044; **(B)** Expression of *ompK36, ompK35*, and *ompR* in NTUH-K2044 incubated with culture medium with 1.0% and 2.5% NaCl. The expression of *ompK36* was used as control in **(A)** while those of *ompK36*, *ompK35*, and *ompR* under 1.0% NaCl were used as controls respectively in **Figure 3B**. One-way ANOVA was used in **(A)** while two-way ANOVA was used in **(B)**. The error bars in figure are standard deviations. **p < 0.01. ***p < 0.001. ****p < 0.0001.

The ‘basal’ expression levels of *ompK35, ompK36, ompK26*, and *ompK37* in NTUH-K2044 were not consistent (**Figure 3A**) with each other: *ompK36* expression was dominant and *ompK3*5 expression was less than 1/50 of that of *ompK36*. The expression of *ompK26* and *ompK37* equaled only about 1/1000 and 1/10000 of that of *ompK36*, respectively.

Compared with the isotonic environment (1.0% NaCl), the expressions of *ompK35* and *ompK36* were found to change in the hypertonic environment (2.5% NaCl). The expression of *ompK35* significantly decreased while the expression of *ompK36* increased (**Figure 3B**). The expression level of *ompR* also increased.

### 3.4 The impacts of OmpK35/36 on the drug-resistance of NTUH-K2044

As shown in **Table 2**, the change of MICs in OmpK36-absent strains were greater than those in OmpK35-absent strains. After the deletion of *ompK36*, the MICs of piperacillin, cefoxitin, cefazolin, cefuroxime, imipenem and meropenem increased to 4, 4, 4, 8, 4, and 2 times respectively, in comparison with those of the wild type NTUH-K2044; after the dual deletions of *ompK35/36*, the MICs of piperacillin, cefoxitin, cefazolin, cefuroxime, imipenem and meropenem reached to 8, 32, 32, 16, 8, and 8 times respectively. Changes were also found in MICs of cefotaxime and cefepime, and some complementated strains showed a recovery in susceptibilities to these antimicrobials. Moreover, OmpK35/36 were found to exert different effects on different β-lactams. Deletion of *ompK36* conferred higher MICs of imipenem and meropenem than that of *ompK35* but no carbapenem-resistance.

### 3.5 The impacts of OmpK35/36 on the drug-resistance of NTUH-K2044+*bla*_KPC-2_

As shown in **Table 3**, the MICs of ceftazidime, cefepime, and aztreonam against Δ*ompK35*+*bla*_KPC-2_ reached to 4, 2, and 4 times respectively in comparison with those against NTUH-K2044+*bla*_KPC-2_. For Δ*ompK36*+*bla*_KPC-2,_ the MICs of ceftazidime, cefoperazone-sulbactam, cefepime and aztreonam achieved 4, ≥4, 4 and 8 times respectively. As to Δ*ompK35/36*+*bla*_KPC-2,_ the MICs were 4, ≥4, ≥64 and ≥32 times respectively. Moreover, it was also observed that the MICs of CZA increased to 2, 2, and 4 times in Δ*ompK35*+*bla*_KPC-2_, Δ*ompK36*+*bla*_KPC-2_, and Δ*ompK35/36*+*bla*_KPC-2_, respectively but were all within susceptibility breakpoint.

## 4 Discussion

As confirmed in the study, OmpK35 and OmpK36 and their corresponding genes exhibited distinct characteristics in *K. pneumoniae.* OmpK35/36 loss is a contributor but not determinant in the formation of carbapenem-resistance without *bla*_KPC-2_, as well as in the formation of CZA-resistance with one *bla*_KPC-2_.

Gene *ompK35/36* was widely present in *K. pneumoniae* strains (**Figure 1A**). Given the high sequence similarity of OmpK35 and OmpK36 (**Figure 2**) in *K. pneumoniae* strains, they may become potential targets for antibacterial drugs and vaccine development. A notable proportion of *K. pneumoniae* strains with defect in *ompK35/36* were found to carry carbapenemase genes (**Figure 1D**). Since this analysis was conducted at the genomic level without assessing transcription or translation, the actual prevalence of such defects may be substantially underestimated. Previous studies have reported that CRKP frequently harbors point mutations, insertions, or truncations in *ompK35/36*, leading to reduced or absent porin expression, which in turn enhances resistance to β-lactam antibiotics (Bongiorno et al., 2023; Hamzaoui et al., 2018; Joshi et al., 2021; O'Neill et al., 2025). The deficiency of OmpK35/36 in *K. pneumoniae*, combined with the production of diverse β-lactamases (including carbapenemases), has posed a great challenge to the therapy based on carbapenems. Although the genomic data from GenBank may be influenced by regional research biases and may not fully reflect actual clinical distribution, a deep survey of *ompK35/36* prevalence can provide a foundational understanding, which is essential for subsequent functional investigation. It is noted that antibiotics and phages drive region-specific diversity of OmpK36 in *K. pneumoniae* and such diversity confers some influence on drug-resistance of *K.pneumoniae (*Cai et al., 2018; Dunstan and Bamert, 2023; Royer et al., 2025a). However, structure variations of OmpK36 were not included in this study.

Functional studies were conducted using the reference strain NTUH-K2044 to elucidate the role of OmpK35/36 in antimicrobial resistance. In this strain, expression level of porin genes varied considerably: *ompK36* was predominant, and *ompK35* was moderately expressed, while *ompK26* and *ompK37* remained largely silent. Under hyperosmotic stress (2.5% NaCl), the expression balance of *ompK35/36* in *K. pneumoniae* shifted, indicating that the expression of porins was regulated by osmotic pressure. It was consistent with the research results of porins in *Escherichia coli*: the two-component system of EnvZ/OmpR could participate in the regulation of bacterial response to external osmotic pressure stimuli, by regulating the expression of OmpF and OmpC (Kenney and Anand, 2020). In the high-osmolarity intestinal environment, *ompK36* expression was favored. Since OmpK35 formed a larger pore than OmpK36 (Sugawara et al., 2016), its downregulation would help bacteria maintain osmotic homeostasis. Clinically, many isolates exhibited a shift from OmpK35 to the smaller-pored OmpK36, reducing antibiotic uptake and enhancing bacterial survival (Bongiorno et al., 2023; Rocker et al., 2020).

Antibiotic susceptibility tests revealed that OmpK35/36 loss primarily affected resistance to β-lactams (**Table 2**). Deficiency in one or both porins increased MICs of various β-lactams (Srinivasan et al., 2012; Asare Yeboah et al., 2025; Birgani et al., 2025). The impact was found more in Δ*ompK36* than in Δ*ompK35*, which was consistent with the dominant expression of OmpK36 (Yasmin et al., 2024) in NTUH-K2044. But if *ompK35* was further deleted, it would result in even higher drug-resistance compared to single mutants, meaning that Δ*ompK35/36* exhibited higher antibiotic resistance than Δ*ompK35* or Δ*ompK36*. For Δ*ompK35/36*, the MICs of cefoxitin, cefazolin, and meropenem reached 8, 8, and 4 times compared to those of Δ*ompK36* respectively. Complementation only partially restored susceptibility, which was likely due to limited OmpK36 re-expression (20 ~ 30% of wild-type levels, see **Supplementary**). The different effect of OmpK35/36 on various β-lactams may stem from difference in antibiotic size, structure and charged properties, which would influence passage through the porin channels (Matovina et al., 2021; Acharya et al., 2024; Royer et al., 2025b). Therefore, significant difference in molecular weights, three-dimensional structures and charged properties among various β-lactams may be the core reasons for the varying degrees of impacts of OmpK35/36 deficiencies on drug-resistance.

Moreover, the loss of OmpK35/36 had a very limited impact on resistance to carbapenems and did not result in the formation of CRKP (**Table 2**). The antimicrobial susceptibility tests of conjugants are shown in **Table 3**. Firstly, it should be emphasized that the interaction between HvKP and drug-resistance plasmids after conjugation is not discussed here. Some literature suggests that HvKP strains carrying *bla*_KPC_-positive plasmids (K1/K2 HvKP strains acquire carbapenem-resistance plasmids) were difficult to simultaneously possess hypervirulence and carbapenem-resistance, and both may decrease when the two were combined (Tian et al., 2022). In this paper, the restoring sensitivity of conjugant NTUH-K2044+*bla*_KPC-2_ to aztreonam, cefepime, and so on, seemed to verify this. However, this study was conducted under the same basic conditions for antimicrobial susceptibility tests of the conjugants, with the only difference being whether OmpK35/36 exists. Then, it can be concluded from **Table 3** that the deletion of OmpK35/36, particularly the double deletion, greatly helped NTUH-K2044+*bla*_KPC-2_ induce resistance to β-lactams. It can be also observed that the absence of OmpK35/36 can increase the MIC of CZA against NTUH-K2044+*bla*_KPC-2_; single OmpK35/36 loss doubled the MIC and double loss quadrupled the MIC, which is different from another study (Nelson et al., 2017). Nevertheless, the MIC of CZA against Δ*ompK35/36*+*bla*_KPC-2_ (1/4 mg/L) was far away from resistance breakpoint (16/4 mg/L). Recent study (Uehara et al., 2025a) investigated the impact of porin deletions on cefepime-taniborbactam activity against *K. pneumoniae* and found taniborbactam didn’t have a strong dependence on OmpK35 or OmpK36 for periplasmic accumulation. In all, the deficiency of porin proteins has a promoting effect on other resistance mechanisms of *K. pneumoniae* but it is not a determinant of carbapenem or CZA resistance.

In summary, the conservation of the *ompK35/36* and OmpK35/36 provided a possible molecular basis for the development of new targeted antibacterial drugs and vaccines, which was beneficial for better prevention and treatment of *K. pneumoniae* infections. The absence of OmpK35/36 could lead to increased resistance of strains to certain β-lactams. Although OmpK36 was the dominant porin, OmpK35 could play a synergistic role in the drug-resistance of *K. pneumoniae* while they were both absent. The absence of OmpK35/36 alone or both cannot determine the formation of carbapenem or CZA resistance, which only plays an auxiliary role in such formation.

## Data Availability

The datasets presented in this study can be found in online repositories. The names of the repository/repositories and accession number(s) can be found in the article/**Supplementary Material**.
